# Predicting Heart Cell Types by Using Transcriptome Profiles and a Machine Learning Method

**DOI:** 10.3390/life12020228

**Published:** 2022-01-31

**Authors:** Shijian Ding, Deling Wang, Xianchao Zhou, Lei Chen, Kaiyan Feng, Xianling Xu, Tao Huang, Zhandong Li, Yudong Cai

**Affiliations:** 1 School of Life Sciences, Shanghai University, Shanghai 200444, China; dingshijian@shu.edu.cn; 2 State Key Laboratory of Oncology in South China, Collaborative Innovation Center for Cancer Medicine, Department of Medical Imaging, Sun Yat-sen University Cancer Center, Guangzhou 510060, China; wangdl@sysucc.org.cn; 3 Center for Single-Cell Omics, School of Public Health, Shanghai Jiao Tong University School of Medicine, Shanghai 200025, China; zhouxch1@shanghaitech.edu.cn; 4 College of Information Engineering, Shanghai Maritime University, Shanghai 201306, China; lchen@shmtu.edu.cn; 5 Department of Computer Science, Guangdong AIB Polytechnic College, Guangzhou 510507, China; kyfeng@gdaib.edu.cn; 6 Guangdong AIB Polytechnic College, Guangzhou 510507, China; xuxl@gdaib.edu.cn; 7 Bio-Med Big Data Center, CAS Key Laboratory of Computational Biology, Shanghai Institute of Nutrition and Health, University of Chinese Academy of Sciences, Chinese Academy of Sciences, Shanghai 200031, China; 8 CAS Key Laboratory of Tissue Microenvironment and Tumor, Shanghai Institute of Nutrition and Health, University of Chinese Academy of Sciences, Chinese Academy of Sciences, Shanghai 200031, China; 9 College of Food Engineering, Jilin Engineering Normal University, Changchun 130052, China

**Keywords:** heart cell, single-cell profiles, machine learning method, biomarker, decision rule

## Abstract

The heart is an essential organ in the human body. It contains various types of cells, such as cardiomyocytes, mesothelial cells, endothelial cells, and fibroblasts. The interactions between these cells determine the vital functions of the heart. Therefore, identifying the different cell types and revealing the expression rules in these cell types are crucial. In this study, multiple machine learning methods were used to analyze the heart single-cell profiles with 11 different heart cell types. The single-cell profiles were first analyzed via light gradient boosting machine method to evaluate the importance of gene features on the profiling dataset, and a ranking feature list was produced. This feature list was then brought into the incremental feature selection method to identify the best features and build the optimal classifiers. The results suggested that the best decision tree (DT) and random forest classification models achieved the highest weighted F1 scores of 0.957 and 0.981, respectively. The selected features, such as NPPA, LAMA2, DLC1, and the classification rules extracted from the optimal DT classifier played a crucial role in cardiac structure and function in recent research and enrichment analysis. In particular, some lncRNAs (LINC02019, NEAT1) were found to be quite important for the recognition of different cardiac cell types. In summary, these findings provide a solid academic foundation for the development of molecular diagnostics and biomarker discovery for cardiac diseases.

## 1. Introduction

The heart is a complex organ containing various cardiac cell types, and the interaction between different heart cell types could realize the important functions of the heart. Previous pioneering studies have shown that the heart is composed of approximately 70% non-cardiomyocytes and 30% cardiomyocytes [1]. Cardiomyocytes could be divided into atrial myocytes and ventricular myocytes, while non-cardiomyocytes mainly include fibroblasts, smooth muscle cells, pericytes, and endothelial cells. These cells form four chambers with different morphologies and functions, and they complete the systemic blood circulation [2].

Cardiomyocytes are responsible for contractile function, and they are the most important part. However, they do not function in isolation. Fibroblasts account for more than 40% of the total cells in the ventricle. Their core function is to maintain the cardiac extracellular matrix homeostasis and provide structural and mechanical support for the cardiomyocytes [3]. The mural cells of the vessel wall are mainly composed of smooth muscle cells and pericytes, and these two cell types are important for vascular integrity and heart function [4]. Endothelial cells form the inner layer of blood and lymphatic vessels; they maintain blood circulation by regulating the permeability and caliber of blood vessels and play an important role in controlling and maintaining the growth, contractility, and rhythm of the heart [5,6]. Mesothelial cells are transitional mesodermal-derived cells with similar morphological and functional characteristics to endothelial cells. They can secrete angiogenic factors, which are important for angiogenesis [7]. Heart adipose tissue not only can supply energy locally but also has heart repair functions, such as new blood vessel formation and immune regulation [8,9]. Immune cells and neurons are also very important for the functional homeostasis [10,11].

Identifying cell components and cell types are important for understanding cell functions, especially in complex organs, where multiple cell types work together. There are two types of traditional methods for cell type annotation: (1) cell marker-based methods, such as CellAssign [12], which needs high quality cell specific expressed genes, but most cell types do not have very specific biomarkers. (2) reference dataset-based methods, such as SingleR [13], which compare the scRNA data with reference scRNA data with known cell types and make predictions. Previous studies have reported some markers for cardiac cells, such as in atria (NPPA and SLN), ventricles (MYL2 and MYL3), endothelial cells (FABP4 and AQP7), smooth muscle cells (ACTA2), fibroblasts (COL1A1), pericytes (PDGFRB) or immune cells (PTPRC), neurons (NEXN1), and adipocytes (GPAM and FASN) [14,15,16,17]. Although these genes are very important for each cell type, the maintenance of cell function depends on the interaction among different genes. Therefore, revealing the specific expression patterns of different cell types, especially the expression features that distinguish them from other cell types, is very important for an enhanced understanding of fate decisions and cell functions.

On the basis of existing single cell profiling datasets from the Human Cell Atlas study of adult human heart cells [17], machine learning algorithms were used in the present study to analyze data to extract gene expression characteristics and biomarkers to characterize different heart cell types. Machine learning algorithms can extract hidden biomarkers that cannot be found by traditional methods through in-depth research on a large number of cell gene expression data. By using the light gradient boosting machine (lightGBM) algorithm [18], a ranking feature list was generated on the basis of the importance of these features. Then, the incremental feature selection (IFS) method [19] with decision tree (DT) [20] and random forest (RF) [21] algorithms was applied to determine the best number of features and build the optimal classifier. As a result, the most relevant gene features and decision rules were identified. Through these rules, 11 cell types could be accurately classified. Meanwhile, the results of Gene Ontology and Kyoto Encyclopedia of Genes and Genomes pathway enrichment analysis suggested that the selected genes may have important significance for the phenotype of cells or function of the heart. Further research on these genes may help clarify the detailed mechanism of heart development. In short, this research identified a group of potential cardiac cell biomarkers and a precise classifier composed of many decision rules, thus providing insights for further research on cardiac development and function. The method we proposed to identify heart cell types was a type of reference dataset-based method. But unlike traditional reference dataset-based methods which use all the genes, we only use discriminative genes selected with feature selection methods. The method had the merits of both two types of traditional methods: (1) we only considered the discriminative genes; therefore, it was faster and more explainable than the refence dataset-based methods; (2) we quantitively considered the combinations of expression levels, i.e., expression rules, rather than only cell specific markers; therefore, it was more accurate than the cell marker-based methods. The following text was organized as follows: (1) Section 2 lists the dataset analyzed in this study and algorithms used in this study; (2) The results were presented in Section 3; (3) Extensive discussion on results was provided in Section 4; (4) Section 5 summarized this study.

## 2. Materials and Methods

### 2.1. Study Design

**Figure 1:**
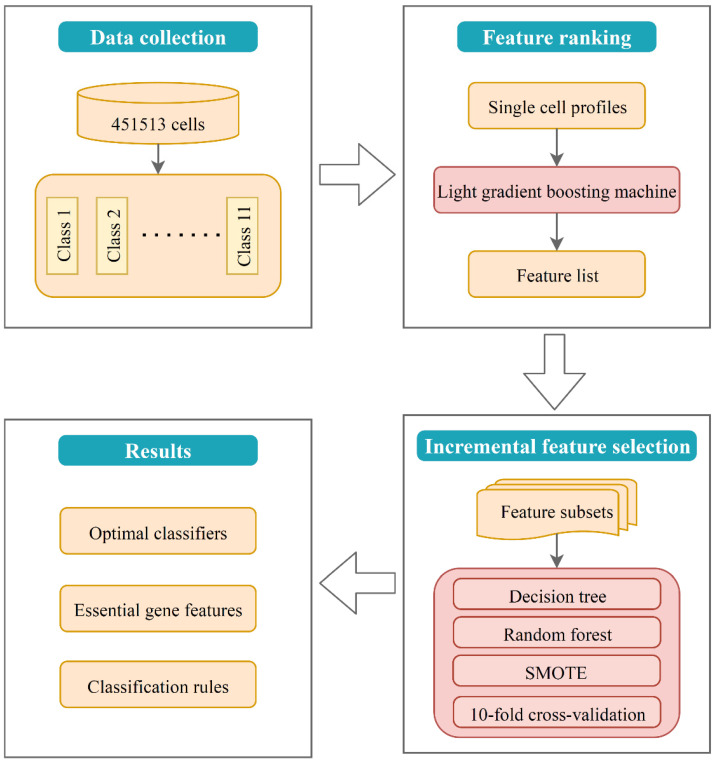
Flow chart of the study design. First, lightGBM method was applied to rank the features of single-cell gene expression profiles into a ranked list. Second, IFS method with machine learning algorithms was used to detect the best number of features and build the optimal classifiers and decision rules. Finally, functional enrichment analysis was performed on the optimal gene feature set.

In this study, the interpretation of the model (i.e., classifier) was consisted of two parts, that is, interpretation on (1) single gene expression signatures and (2) combined gene expression rules. Single-gene interpretation explained the effect of single optimal genes on the classification, while combined-gene interpretation focused on exploring how multiple genes contribute to the classification together. The whole framework of this study is shown in Figure 1.

### 2.2. Data Collection

Raw dataset was downloaded from the publicly available Human Cell Atlas Data Coordination Platform, with accession number: ERP123138 (https://www.ebi.ac.uk/ena/ browser/view/ERP123138, accessed on 29 January 2021) [17]. The processed 10X Genomics dataset included the expression profiles of 33,538 genes in 451,513 cells from 11 cell types. The 11 major cell types and the corresponding sample sizes are presented in Table 1.

### 2.3. Feature Ranked by LightGBM

LightGBM is a well-known boosting learning machine [18] that combines many weak classifiers to achieve a single strong one. It could be regarded as an improved version of gradient boosting DT (GBDT) [22], which recurrently fits a new DT by using the negative gradient of the loss function of the current DT as the approximate value of the residual. The main differences between lightGBM and gradient-based one-side sampling (GOSS) lie in the two new strategies adopted by lightGBM. These are GOSS and exclusive feature bundling (EFB), which both greatly improve the efficiency and ensure the accuracy of classification. In GBDT, the gradient of a sample in calculating the residual error of a DT reflects the contribution of the sample to subsequent classification. Therefore, GOSS down samples the training data by randomly screening out most of the samples with small gradient and keeping a small number of them to maintain the distribution of the data. EFB bundles the mutually exclusive features together to reduce the dimension of the data. The mutually exclusive features are those that rarely take nonzero values simultaneously, and no or very little information is lost by bundling them as a new feature. EFB is realized by solving a graph coloring problem with the use of a greedy algorithm with a constant approximation ratio. As described in LightGBM’s documentation (https://lightgbm.readthedocs.io/en/latest/, accessed on 10 May 2020), the advantages of lightGBM include faster training efficiency, low memory usage, higher accuracy, support for parallel learning, and being able to handle large-scale data. In addition to classification, lightGBM sorts features in accordance with their importance, which is quantified by the number of times the feature is selected to build DTs. The more times a feature is used, the higher the ranking. In this research, the features were sorted using lightGBM for further analysis. The lightGBM program was implemented through a Python module.

### 2.4. IFS Method

Once the feature list *F* was generated by sorting the gene features with the lightGBM method, the number of significant features could still not be determined. Here, the optimal number of features was discovered using the IFS method [19]. IFS first generates a series of feature subsets from the feature list *F* on the basis of the specific step size. For example, when the step size is 5, the first feature subset *f_1_* generated is the top five features in *F*, the second feature subset *f_2_* is the top 10 features in *F*, and so on. Next, each classifier trains on a training set whose samples are expressed by the features in a feature subset. The performance of the classifier is evaluated using 10-fold cross-validation [23] and synthetic minority oversampling technique (SMOTE) [24], and the classifier with the best performance is considered the optimal classifier, and its trained subset is regarded as the optimal subset.

### 2.5. Classifier Building with DT and RF

In this study, DT and RF were adopted to build classifiers. Their descriptions are as follows:

RF [21,25,26,27,28] is a classification algorithm that integrates multiple DT classifiers. Through a bootstrap resampling technique, a new training set is composed, and the DT is constructed by randomly selecting samples and features from the original data set. The prediction labels of RF are obtained by combining the prediction results of multiple DT classifiers by using the principle of minority rule. RF has few parameters to tune, users can only choose a proper number of DTs so that it can yield good performance. Because RF contains several DTs, it has excellent noise tolerance and can avoid the overfitting problem. Importantly, although DT is a relative weak classifier, RF is much stronger. Thus, it is widely used in omics research.

Through RF, investigators can build an efficient classifier. However, it is a black-box classifier. Its classification principle is not easy to understand. Accordingly, we cannot extract essential difference of heart cells in different types. In view of this, DT [20,29,30] was also employed in this study, which is widely used in the field of biomedical research, because the decision rules generated by DT can effectively elucidate how decisions are made for classification or regression tasks. In another word, a DT is a white-box classifier that splits the data many times on the basis of certain thresholds in the features by using the IF-THEN format. These IF-THEN formats constitute decision rules, which can clearly exhibit special patterns for each class. In this study, these patterns indicated specific characteristic of heart cell types. Although DT provides relative low performance, it can give novel insights to study heart cells. In the output file of DT, the value of “passed counts” indicates the number of samples satisfying the condition of the rule. The above two algorithms were performed by the scikit-learn program with default parameters in Python [31].

### 2.6. SMOTE

The different number of samples from different cell types leads to the problem of data imbalance. Synthetic minority oversampling technique (SMOTE) was applied to minimize the effect of sample imbalance on the construction of classifiers [24]. It generates many synthetic samples for minority cell categories on the basis of the principle of k-nearest neighbors [32]. For each cell type, except for the cell type with the highest number of samples, new synthetic samples were added to this cell type via SMOTE until the number of samples of each type was almost the same. The SMOTE program was accessed from https://github.com/scikit-learn-contrib/imbalanced-learn (accessed on 24 March 2021), and the parameters were set to default.

### 2.7. Performance Measurement

As the number of samples in each category in the dataset could be strongly unbalanced, the weighted *F_1_* score [33,34,35] is an appropriate measurement of the classifier’s performance. First, the *F_1_* score was calculated using the following formula:

(1) $$F_{1}\mathrm{score}=\frac{2\times precision\times recall}{precision+recall}$$

Next, weighted average was performed to the *F*_1_ score of each category, and the weight was the proportion of the number of categories in the correct label. This measurement was called weighted *F*_1_ score.

### 2.8. Functional Enrichment

For functional enrichment analysis, the Clusterprofile package was applied to the annotation of Gene Ontology (GO) and Kyoto Encyclopedia of Genes and Genomes (KEGG) pathway, where *p* < 0.05 was taken as the screening criterion [36]. The GO terms are divided into three subgroups, namely, biological process (BP), cellular component (CC), and molecular function (MF).

## 3. Results

### 3.1. Results of LightGBM Method on the Dataset

In this study, single cell expression profiles of 451,513 cell samples and 11 cell types for heart disease were obtained, and each sample of heart cell type was represented by the expression of 33,537 genes. LightGBM method was first applied to rank the genes into a feature list on the basis of feature importance to filter out the important set of features from these genes, and the results are provided in Table S1. The top 20 genes in the list are shown in Table 2.

### 3.2. Results of IFS Method with RF

A feature ranking list was obtained by the lightGBM method, but the optimal number of features was not determined. The IFS method was applied to optimize the selected gene features. We first adopted RF to execute IFS method. The propuse was to construct an efficient classifier for classifying heart cells.

**Figure 2:**
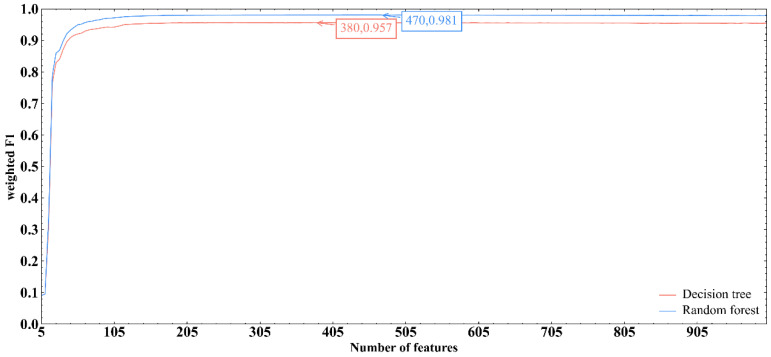
IFS curve of DT and RF methods. IFS curves were plotted, with the number of features as X-axis and the performance as Y-axis. The highest weighted F1 scores generated by RF and DT were marked.

**Figure 3:**
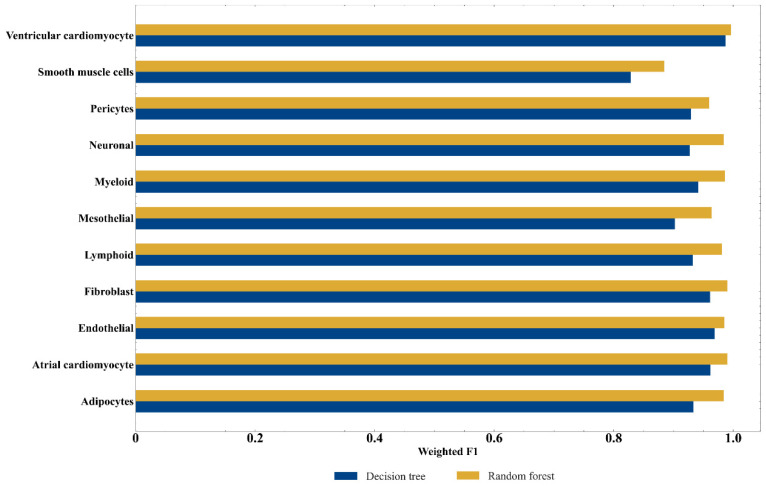
Performance of two optimal classifiers on each cell type.

Based on the feature list provided in Table S1, the IFS method produced a series of feature subsets with the step size of 5. A SVM classifier was then built based on each feature subset to predict the label of each sample. To save time, we only considered top 1000 gene features in the list. The evaluation results of all SVM classifiers are shown in Table S2. The IFS curve with the number of features as the X-axis and the performance of each classifier, measured by weighted F1score, as the Y-axis is drawn in Figure 2. RF obtained the optimal weighted F1 score of 0.981 with the top 470 features. Accordinly, the optimum RF classifier can be built using these 470 features. Table 3 provides the detailed evaluation metrics of the optimal RF classifier. It also provided good performance on other metrics. In addition, the F1 score for each cell type under the optimal RF classifier is presented in Figure 3. Such optimum RF classifier showed excellent performance on the prediction of each category, indicating the effectiveness of this classifier.

**Figure 4:**
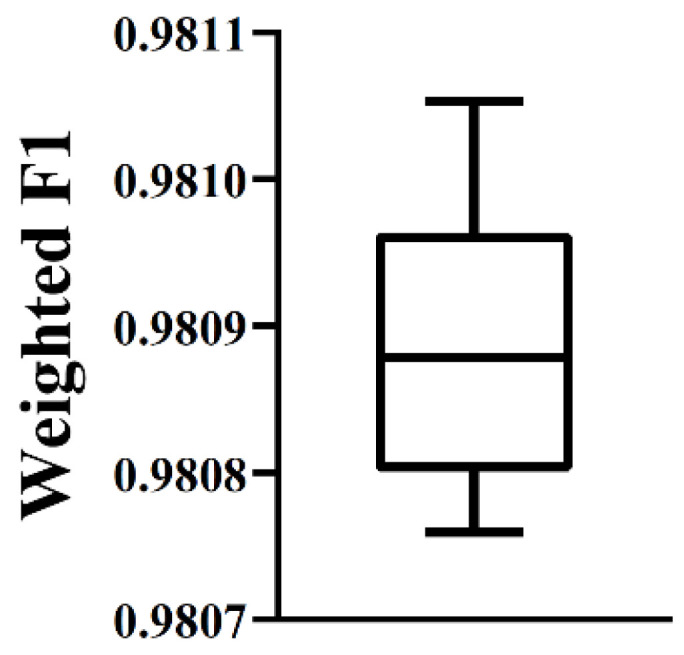
Box plot to show the performance of the optimal RF classifier on datasets with noise. The performance is almost same as that on the original dataset, proving the robustness of the optimal RF classifier.

As mentioned above, the optimal RF classifier provided quite good performance. It can be an efficient tool to classify heart cells. To further elaborate its robustness, the following test was conducted. First, some noise was added to the dataset, in which cells were represented by features used in the optimal RF classifier. In detail, we randomly selected 10% cells. Each feature of these cells randomly increased or decreased by a small number. On such dataset, the optimal RF classifier was evaluated by 10-fold cross-validation. Above procedures were executed for ten times, producing ten weighted *F*_1_ scores. A box plot was drawn (Figure 4) to show these values. It can be observed that the performance on datasets with noise was almost same as that on the original dataset. This proved the robustness of the optimal RF classifier.

### 3.3. Results of IFS method with DT

Although the optimal RF classifier exhibited quite good performance, it is a black-box classifier that fails to explain the decisions. To extract more insights for the study of heart cells, DT, a white-box algorithm, was employed. We conducted the same procedures on DT that had been done for RF. The performance of DT classifiers on different feature subsets is listed in Table S2. An IFS curve was also plotted, as shown in Figure 2. It can be observed that the highest weighted F1score was 0.957 when top 380 features were used. Thus, an optimum DT classifier can be built with these features. Other metrics of such classifier are provided in Table 3. The performance of such classifier on all cell types is shown in Figure 3. Evidently, the performance of the optimum DT classifier was lower than that of the optimum RF classifier, which conform to the general fact that RF is more powerful than DT. However, DT has its own merit that RF cannot have. The classification procedures of DT were completely open, which make us possible to understand its classification principle, thereby giving new insights to uncover the difference of heart cells in different types.

### 3.4. Classification Rules Generated by the Optimal DT Classifier

**Figure 5:**
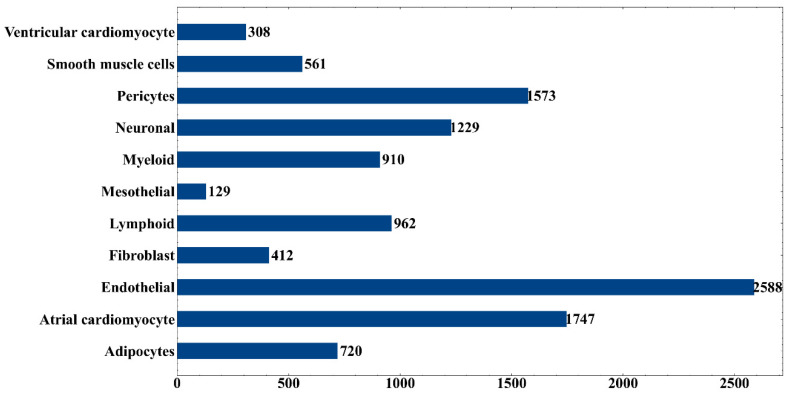
Number of rules produced by the optimal DT classifier on each cell type.

The optimum DT classifier was built based on top 380 features. Accordingly, we used these features to represent all heart cells. Such representation of all heart cells was learnt by DT. A large tree was obtained, from which we constructed 11,139 interpretable rules. The detailed rules are listed in Table S3. The number of rules for each category is shown in Figure 5. Endothelial cells obtained the largest number of rules, with 2588, followed by atrial cardiomyocytes and pericytes. A detailed description of these rules could be found in the Section 4.2.

### 3.5. Functional Enrichment Analysis with Optimal Gene Set

**Figure 6:**
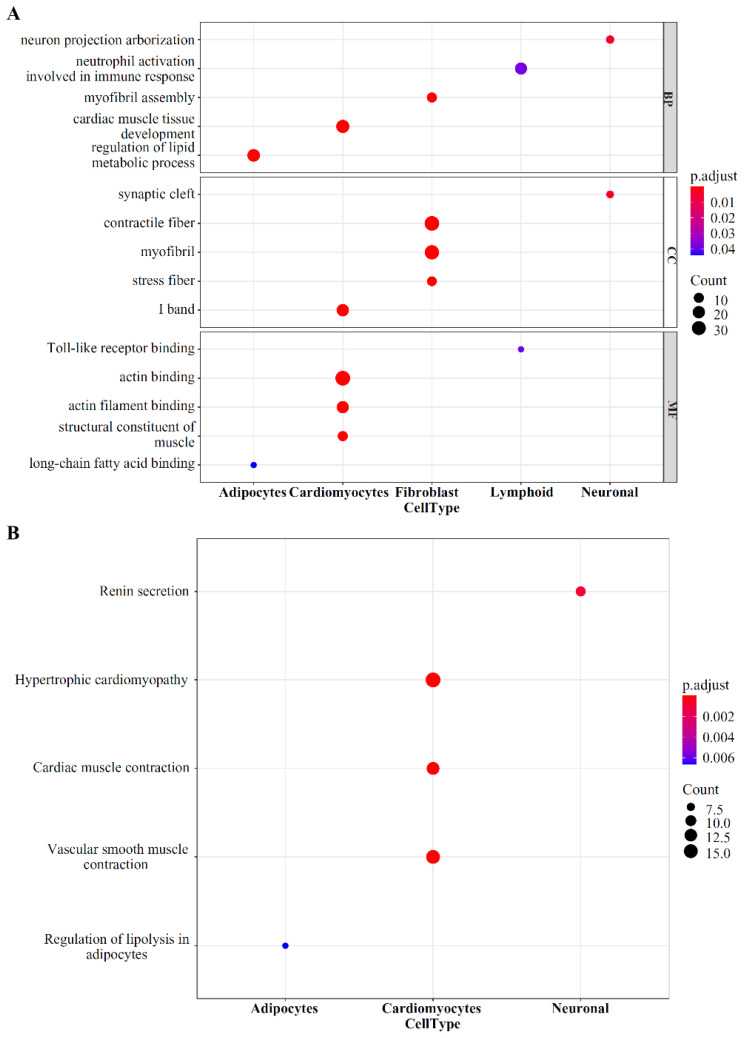
GO term and KEGG pathway analysis for the top 470 genes. (**A**) Top 15 key GO terms. (**B**) Top five key KEGG pathways.

The best gene set was obtained, including the top 470 features, by using the IFS method. These genes were analyzed by GO and KEGG pathway functional enrichment, and the results are presented in Table S4 and Figure 6. Many genes were enriched in the KEGG pathway of hypertrophic cardiomyopathy and dilated cardiomyopathy, and some genes were enriched in the BP of heart process, indicating that these genes may be associated with the development of heart disease and further demonstrating the effectiveness of the method.

## 4. Discussion

In this study, several machine learning algorithms were applied to classify different cell types from single-cell and single-nucleus transcriptome profiles of six adult heart regions. First, the lightGBM method was performed to obtain a ranking feature list in accordance with feature importance. Second, an RF algorithm was applied to construct a precise classifier with a high classification accuracy of 0.981. However, as a black-box classifier, this classifier cannot reveal the different expression patterns of different heart cell types. Therefore, the DT algorithm was further used to obtain a group of decision rules. By using the top 380 features, different cell types could be distinguished, with a high classification accuracy of 0.957. Furthermore, novel biomarkers or expression patterns may be identified by analyzing the expression pattern of 310 selected genes within these decision rules.

### 4.1. Candidate Gene Expression Features Discriminating Different Heart Cells

A total of 470 selected features (genes) were used to classify 452,136 heart cells into 11 cell types. Among them, the top ranked genes are usually more decisive for distinguishing different cell types. Some relevant experimental evidence that supported the results were presented.

NPPA (ENSG00000175206) encodes natriuretic peptide A (ANP), which is highly expressed in the heart muscle and related to the control of extracellular fluid volume and electrolyte homeostasis. Studies have found that NPPA is expressed primarily in the heart, and atria have higher expression than ventricles. NPPA can regulate vasodilation, natriuresis, diuresis, and aldosterone synthesis and further influence blood pressure. Moreover, it is involved in inhibiting cardiac hypertrophy, cardiac fibrosis, and cardiac remodeling by inducing cardiomyocyte apoptosis and attenuating the growth of cardiomyocytes and fibroblasts [37]. In adipocytes, ANP can promote white adipocyte browning to increase energy expenditure via a PKG-p38 mitogen-activated protein kinase mediated pathway and make the heart as a central regulator of adipose tissue biology [38]. These findings are consistent with the results of the present study, which showed that NPPA has a crucial role in different functional heart cells.

Similarly, gene LAMA2 (ENSG00000196569), which encodes the laminin subunit, plays an important role in normal heart function. Studies have demonstrated that homozygous mutation of LAMA2 can cause unstable myotube formation in various cardiac muscle, and abnormal LAMA2 expression may lead to heart dieases, such as cardiomyopathy, heart failure, and dilated cardiomyopathy [39,40]. A previous adult human heart research also showed that LAMA2 has different expression levels in fibroblasts, cardiac adipocytes, and other cell types [17].

Other key features in our results are also important for cardiac function. For example, the gene DLC1 (ENSG00000164741) is highly expressed in endothelial cells and a small number of ventricular cardiomyocytes [17,41]; RYR2 (ENSG00000198626) encodes a calcium channel component associated with cardiomyocyte and smooth muscle cell contraction and thermogenesis in beige adipocytes [42,43,44]; TTN (ENSG00000155657) encodes titin, a large abundant protein of striated muscle, mainly found in cardiac and skeletal human muscle. Mutations in these genes may cause a variety of cardiac diseases [45,46,47].

More importantly, we found that some long non-coding RNAs (lncRNA) are important for differentiating cardiac cell types. For example, LINC02019 (ENSG00000273356), it is the top gene in our feature ranking. The product of this gene is a long intergenic non-protein coding RNA (lincRNA). We used the starBase tool to study its related RNA-binding proteins [48]. The most related proteins include EIF4A3, ELAVL1, LIN28A. Studies have shown that EIF4A3 is associated with acute myocardial infarction, and knockout of EIF4A3 can lead to failure of heart looping [49,50]. ELAVL1 plays an important role in inhibiting hyperglycemia-induced cardiomyocyte pyroptosis and regulating ferroptosis in myocardial injury [51,52]. LIN28A is also implicated in various cardiac injuries or diseases [53,54].

NEAT1 (ENSG00000245532) produces a lncRNA that may act as a transcriptional regulator for numerous genes. NEAT1 was markedly downregulated in cardiomyocytes following ischemia–reperfusion-induced injury. Moreover, by interacting with microRNA-125a-5p, NEAT1 could modulate the concentration of BCL2L12, which in turn regulates cardiomyocyte apoptosis [55]. Other studies also found that NEAT1 may influence myocardial injury repair through the MAPK and TLR2/NF-κB signaling pathways [56,57].

In summary, these genes are all related to cardiac structure and function, and they show various expression levels in the ventricle, atrium, and other cell types. Therefore, these genes could be used as decisive feature for distinguishing different cardiac cells, and we also confirmed that some lncRNAs may have more specific roles in the maintenance of normal cardiac function.

### 4.2. Candidate Gene Expression Rules Discriminating Different Heart Cells

Through DT method, a classifier consisting of 11,139 decision rules involving 380 selected features was built. Each cell type was assigned some rules, as shown in Figure 4. According to the value of “passed counts”, top three rules for each cell type were extracted and listed in Table 4. The genes involved in these 33 rules were analyzed in combination with the existing literature to prove the reliability of the results. Studying other rules may also help find some new characteristics of cardiac cell subtypes, which may provide new insights into the in-depth understanding of cardiac development and function.

#### 4.2.1. Cardiomyocytes

Cardiomyocytes generate contractile force; thus, they normally show high expression of sarcomere proteins and calcium-mediated processes [17]. The present study showed that atrial cardiomyocytes and ventricular cardiomyocytes highly expressed TTN, which was the most important factor for distinguishing cardiomyocytes and non-cardiomyocytes. As mentioned above, atria showed higher expression of NPPA than ventricles [58]. The decision rules in the present study also showed the same expression pattern. In addition, atrial cardiomyocytes required a higher expression of KCNJ3 (ENSG00000162989) and MYL7 (ENSG00000106631) than ventricular cardiomyocytes. KCNJ3 encodes an integral membrane protein and an inward-rectifier type potassium channel. Studies have found that KCNJ3 plays an important role in governing cardiac electrical activity, and atrial cardiomyocytes have high KCNJ3 levels than ventricular cardiomyocytes [59]. Other studies also demonstrated that the protein encoded by MYL7, which is a part of myosin, is highly expressed in atrial cardiomyocytes [60].

#### 4.2.2. Fibroblasts and Vascular, Stromal, and Mesothelial Cells

In the decision rules, smooth muscle cells showed higher expression of MYH11 (ENSG00000133392) than other cell types. MYH11 encodes myosin heavy chain 11, and high MYH11 level is a marker of mature contractile phenotype of smooth muscle cell, while downregulation or mutation of MYH11 is associated with vascular disease [61]. ACTA2 (ENSG00000107796) is also a marker of smooth muscle cells; it is known as smooth muscle α actin, which is usually highly expressed in smooth muscle cells, pericytes, and myofibroblasts [62]. Studies found that mutation of ACTA2 could cause coronary artery disease and thoracic aortic disease. An experimental study on smooth muscle cells and myofibroblasts harboring ACTA2 mutations indicated that occlusive disease is associated with increased proliferation of smooth muscle cells [63,64].

ABCC9 (ENSG00000069431) encodes a protein that is a member of the ATP-binding cassette transporter superfamily. In this research, the decision rules of pericytes showed high ABCC9 expression requirement. This finding is in accordance with various published studies, which showed that ABCC9 is highly expressed in pericytes and could be a biomarker for it [17,65,66].

VWF (ENSG00000110799) was required to be highly expressed in endothelial cells in the decision rules. As a protein coding gene, VWF encodes a glycoprotein involved in hemostasis, and it is reported to be highly expressed in endothelial cells in human cells [67], thus confirming the results of the present study.

Unlike endothelial cells, smooth muscle cells, pericytes, and mesothelial cells, fibroblasts are not involved in the construction of basement membranes. As known, collagen IV, laminin-entactin/nidogen complexes, and proteoglycans are the major molecular constituents of basement membranes [68]. This information was confirmed in the decision rules because LAMA2 (ENSG00000196569) and CD36 (ENSG00000135218) showed a relatively low expression in fibroblasts. As CD36 encodes collagen IV and LAMA2 encodes a subunit of laminin, they both required to be highly expressed in other four cell types.

Although existing studies could not confirm why mesothelial cells need to relatively highly express PLA2G2A in the rules, this may be a coincidence caused by the limited number of mesothelial cells or the unknown effect of PLA2G2A on mesothelial cells. Similarly, other types of cells have some very meaningful genes that have not been studied in depth. However, the results of this study showed that they may be important.

#### 4.2.3. Adipocytes and Immune and Neuronal Cells

Adipocytes, immune cells, and neuronal cells showed low expression of sarcomere proteins or basement membranes components. NRXN1 (ENSG00000179915) encodes neurexin 1, which is a cell surface receptor involved in the formation of synaptic contacts, and efficient neurotransmission depend on NRXN1 [69]. The same expression pattern could be observed from the decision rules of neuronal cells. Another highly expressed gene in the rules was neuronal growth regulator 1 (NEGR1, ENSG00000172260). NEGR1 mediates neural cell communication and synapse formation, and its downregulation is related to obesity, learning difficulties, intellectual disability, and psychiatric disorders [70]. Thus, appropriate NEGR1 expression is necessary to maintain neuronal cell function.

Hematopoietic cells are commonly classified into myeloid and lymphoid cells. The expression of CD163 (ENSG00000177575) is the main criterion used to distinguish between myeloid and lymphoid cells in the rules. As CD163 encodes a member of the scavenger receptor cysteine-rich superfamily, it is exclusively expressed in monocytes and macrophages [71]. Thus, CD163 could serve as a marker for myeloid cells, and this finding also confirmed the results of the present study.

Adipocytes showed high PLIN1 (ENSG00000166819) and ACACB (ENSG00000076555) levels than other cells. PLIN1 and ACACB participate in the inhibition of lipolysis and the regulation of fatty acid oxidation, respectively. According to the recent publications, these two genes are essential for the maintenance of adipocyte functions [72,73].

In conclusion, the genes that are highly expressed in the decision rules are often markers of the cell types or essential for maintaining cell function, which reflects the reliability of the research results. Some genes that are meaningful for cell classification, which have not been investigated in detail before, may have important implications for the function of the corresponding cell type.

### 4.3. Functional Analysis of the Optimal Gene Set

We also performed GO and *KEGG* pathway enrichments in the 470 decisive features identified by the RF method, and here we presented the relevant enrichment results of some cell types.

The enrichment terms related to adipocytes include GO:0019216 (regulation of lipid metabolic process), GO:0036041 (long-chain fatty acid binding) and hsa04923 (Regulation of lipolysis in adipocytes), which reflect the energy supply and regulation of adipocytes. Similarly, there are also some terms related to lymphoid cells and neurons in the enrichment results, such as GO:0140058 (neuron projection arborization) and GO:0035325 (Toll-like receptor binding).

The primary function of the heart is to effectively pump blood to the body tissues through the contraction–relaxation cycle of myocytes, and the heart is mainly composed of cardiomyocytes and fibroblasts. In our GO and *KEGG* enrichement results, many of the results are related to these two types of cells. Fibroblast-related terms are mainly related to fibers, such as GO:0043292 (contractile fiber), GO:0030016 (myofibril), and GO:0030017 (sarcomere). Sarcomere is composed of a segment of myofibril between two adjacent Z discs, and it is the contractile unit of myofibrils. Studies have found that various genetic mutations in the cardiac sarcomere could lead to defects in sarcomere production and further lead to ventricular dilatation and cardiac dysfunction [74]. The enrichment terms of cardiomyocytes mainly include GO: 0048738 (cardiac muscle tissue development), GO: 0003779 (actin binding) and GO: 0008307 (structural constituent of muscle). Actin plays an essential role in the assembly of cardiac myofibrils, and it is strongly associated with muscle contraction. In the process of myofibrillogenesis, actin is assembled into highly ordered mature state, and the abnormal expression of actin dynamic-related genes usually leads to myofibril abnormalities and heart defects [75]. Meanwhile, the *KEGG* pathway annotation showed that the features are related to muscle contraction and various cardiomyopathies (*KEGG*: hsa05410, hypertrophic cardiomyopathy; *KEGG*: hsa04260, cardiac muscle contraction). The main cause of cardiomyopathy is muscle cell dysfunction caused by genetic variation, especially the dysfunction of genes related to the cytoskeleton–sarcomere connection [76,77]. This further illustrates the important role of these genes in cardiac muscle cell function.

In this part, an extended description about the decisive features was discussed. These terms have been shown to be associated with multiple cardiac cell types, further demonstrating the importance of our decisive features to the function of cardiac cell populations and supporting the reliability and effectiveness of their usefulness in constructing classifiers.

### 4.4. Limitations of This Study

There are several limitations of this study: (1) The data we analyzed was from Human Cell Atlas and it was not known how representative the Human Cell Atlas data is. When more data from various heart locations becomes available, we need to test the model on more data. (2) The tissue was only from adult human hearts. Can the model be applied to children or infants? Do they have different cell types? We need to compare the adult heart data with child and infant data. (3) The 10X Genomics data was sparse. If the cells were measured with smart-seq, will other low expressed biomarkers and rare cell types be discovered? There may be more cell markers and cell types than the identified ones.

## 5. Conclusions

The feature selection method and machine learning algorithms were applied to build a workflow to determine the essential gene features and specific gene expression rules for classifying different heart cell types. The positive results received from this study indicated that the developed classification models achieved an excellent classification performance. The selected key genes and decision rules were demonstrated to be associated with cardiac structure and function in recently published literature and enrichment analysis. In the future, we will collect data from different heart locations, different age groups and different sequencing platforms to get robust heart cell type annotation model. With the knowledge of gene expression patterns at single cell resolution, we can decipher the cell composition changes for diagnosis and target the dysfunctional cell without harming other cells for precision treatment.

## Figures and Tables

**Table 1:** Sample size of each heart cell type on single-cell dataset.

Index	Cell Type	Sample Size
1	Adipocytes	3799
2	Atrial cardiomyocyte	23,483
3	Endothelial	100,579
4	Fibroblast	59,341
5	Lymphoid	17,217
6	Mesothelial	718
7	Myeloid	23,028
8	Neuronal	3961
9	Pericytes	77,856
10	Smooth muscle cells	16,242
11	Ventricular cardiomyocyte	125,289

**Table 2:** Top 20 genes in a feature list, as ranked by lightGBM method.

Index	Gene Symbol	Index	Gene Symbol
1	LINC02019	11	LAMA2
2	CAMSAP3	12	NPPA
3	AC128685.1	13	LINC01958
4	AL139125.1	14	LMNTD2
5	AL024508.2	15	AC131009.2
6	AL121772.1	16	DLC1
7	LINC02346	17	AC020978.5
8	GLB1L3	18	RYR2
9	C22orf15	19	LDB2
10	UPK3A	20	SPARCL1

**Table 3:** Performance of the two optimal classifiers.

Classification Algorithm	Number of Features	ACC	MCC	Macro F1	Weighted F1
Random forest	470	0.981	0.977	0.973	0.981
Decision tree	380	0.957	0.945	0.934	0.957

**Table 4:** Number and passed counts of the selected 33 rules based on the first three highest passed counts in each cell type.

Rule Index	Cell Type	Passed Counts ^a^	Rule Index	Cell Type	Passed Counts
Rules_4	Atrial cardiomyocyte	14,567	Rules_12	Endothelial	2992
Rules_15	Atrial cardiomyocyte	2451	Rules_159	Mesothelial	199
Rules_25	Atrial cardiomyocyte	1657	Rules_269	Mesothelial	96
Rules_0	Ventricular cardiomyocyte	95,879	Rules_287	Mesothelial	89
Rules_13	Ventricular cardiomyocyte	2856	Rules_20	Neuronal	1950
Rules_14	Ventricular cardiomyocyte	2728	Rules_182	Neuronal	165
Rules_2	Fibroblast	32,635	Rules_189	Neuronal	159
Rules_9	Fibroblast	6595	Rules_36	Adipocytes	1227
Rules_19	Fibroblast	2219	Rules_52	Adipocytes	866
Rules_11	Smooth muscle cells	3115	Rules_81	Adipocytes	486
Rules_17	Smooth muscle cells	2242	Rules_20	Neuronal	1950
Rules_29	Smooth muscle cells	1565	Rules_182	Neuronal	165
Rules_3	Pericytes	21,300	Rules_189	Neuronal	159
Rules_8	Pericytes	7142	Rules_5	Lymphoid	9681
Rules_10	Pericytes	4448	Rules_24	Lymphoid	1673
Rules_1	Endothelial	62,186	Rules_78	Lymphoid	503
Rules_7	Endothelial	8820			

^a^: “passed counts” indicates the number of samples satisfying the condition of the rule.

## Data Availability

The data presented in this study are openly available in Human Cell Atlas Data Coordination Platform at https://www.ebi.ac.uk/ena/browser/view/ERP123138 (accessed on 29 January 2021), reference number [15].

## Supplementary Materials

The following supporting information can be downloaded at: https://www.mdpi.com/article/10.3390/life12020228/s1, Table S1: Ranking results of features, as obtained using lightGBM method; Table S2: Evaluation of the performance of each classifier with different numbers of features by using IFS method with 10-fold cross validation; Table S3: Decision rules generated by DT classifier when using the top 380 features; Table S4: Results of Gene Ontology (GO) and Kyoto Encyclopedia of Genes and Genomes (KEGG) pathway enrichment analysis for the top 470 genes.
